# Ethyl Chloride: A Few Practical Remarks

**Published:** 1904-09

**Authors:** A. L. Flemming

**Affiliations:** Anæsthetist to the Bristol Royal Hospital for Sick Children and Women; Assistant Anæsthetist, Bristol Royal Infirmary


### ETHYL CHLORIDE: A FEW PRACTICAL REMARKS.

BY

A. L. FLEMMING,

*Anæsthetist to the Bristol Royal Hospital for Sick Children and Women; Assistant Anæsthetist, Bristol Royal Infirmary.*

---

WHEN considering the use of ethyl chloride as a general anæsthetic one cannot help feeling surprised that the profession has not before this decided more definitely as to the safety and utility of the drug. But an explanation may probably be found in the great variety of methods used for its administration; for although it *may* be given by means of almost any of the ordinary anæsthetic apparatus, yet there is probably no anæsthetic which, for success, depends upon the way in which it is given so markedly as does ethyl chloride. From a practical point of view the most important points for consideration are: the apparatus, the method of administration, the choice of suitable cases, and the clinical and physiological signs of overdose.

*Apparatus.*—This may be dismissed in a very few words; for although many special inventions have been introduced, still a quite efficient inhaler exists in a Clover's face-piece and bag, connected together without the ether chamber. If different forms of inhalers are tested systematically, and the results compared, it will be found that the advantage, especially in the direction of economy, will lie with one of the forms in which the ethyl is sprayed on to lint, provided that the lint is renewed frequently and not allowed to become sodden and frozen.

*Administration.*—This is important. Faulty administration is the commonest cause of struggling, and of what may be termed an uncomfortable anæsthesia. Overdosing and mechanical obstruction to the breathing, such as is caused by the falling back of the tongue, and careless selection of subjects, are the chief sources of danger to the patient. It must be borne in mind that whereas nitrous oxide may be given without oxygen, this does not obtain in the case of ethyl chloride, which should always have a certain proportion of oxygen given with it; and this may be effected by commencing with the bag empty and the apparatus charged with the required dose of the drug, the patient being made to expire the first four or five breaths into the bag before the final close application of the face-piece. At the same time, by gradually approximating the apparatus to the face, these four or five inspirations may be used for the insidious introduction of the vapour to the patient. The bag will now contain vaporised ethyl chloride and the air of four or five expirations, or, in other words, ethyl chloride vapour mixed with sufficient oxygen for sixteen or twenty respirations, and, as Vernon Harcourt puts it, the patient will be subjected to an atmosphere of the drug rather than to a stream of it. The management of these first four or five breaths is important, and if skilfully carried out will obviate to a great extent any tendency to general struggling or to spasm of the glottis. The admixture of oxygen undoubtedly diminishes the frequency of vomiting. The most reliable signs of anæsthesia are stertorous breathing and rotation downward (or upward), with fixation, of the eyeballs. The stertor is soft, and, in order that he may hear it easily, the anæsthetist must insist on silence being maintained in the theatre. The dose should be as small as possible, and should be very carefully measured by means of the graduated scale on the tube. For patients under 6 years 1 cm. generally suffices; between 6 years and 12 years, 2 cm.; between 12 years and 18 years, 3 cm.; for adults and muscular subjects, 4 cm.; for known strugglers 6 cm. may be used. It is well to remember that after removal of the inhaler narcosis continues to deepen for a few seconds. As is the case with chloroform, the effect of an overdose is aggravated by any interference with the supply of oxygen, and a dose which would otherwise be safe becomes dangerous in the presence of obstructed breathing. For this reason the use of ethyl chloride in operations upon adenoids and tonsils is to be considered safe only when the surgeon will allow the pharynx to be kept sufficiently clear to permit of practically uninterrupted breathing. An understanding to this effect should exist between the surgeon and the anæsthetist. Every patient should be prepared as regards the viscera and the loosening of clothes. Prolonged anæsthesia often gives rise to headache in adults, but is free from unpleasant symptoms in children. Repeated administrations are not to be entertained except after intervals of at least half an hour. Swallowing must be avoided as much as possible.

*The Choice of Suitable Cases.*—Most people seem to be able to take this anæsthetic; and some, such as young children, very feeble persons, stout or emphysematous subjects, undoubtedly take it better than they take ordinary gas. The writer has administered it, without noticing any ill effects, to patients suffering from exophthalmic goître, chronic nephritis, diabetes, epilepsy, mitral regurgitation (without loss of compensation), and retroverted gravid uterus. As an "introducer" for ether or chloroform it is excellent. Bad subjects are found in hysterical women, habitual drinkers, and heavy smokers. As regards the subjects of diabetes or exophthalmic goitre, they should not be anæsthetised except when absolutely necessary.

*Overdose.*—Both the immediate and the after-effects of overdose are greatly increased by any interference with the supply of oxygen. In a severe case the patient becomes pale, with widely-dilated pupils, absence of respiratory movements, complete flaccidity of limbs, and has no perceptible radial pulse. Artificial respiration is followed by recovery within a very short time. The after-effects of too much ethyl chloride and too little air are headache, persistent suffusion of the conjunctivæ, pallor, vomiting, abdominal pain, weakness, and a feeling of wretchedness. These symptoms, or some of them, may last for many hours. By a careful regard to the dose used and to the freedom of respiration, accidents and undesirable symptoms seem to be avoidable. The few deaths which have occurred (four have been reported) have been in subjects who were not fit for any anæsthetic, *e.g.* a baby with laryngeal diphtheria, an adult with advanced valvular disease of the heart, &c.

The writer has examined the blood from cases of overdose in human beings, and from cats which have been poisoned with the drug. A curious phenomenon was obtained in nearly every case. The spectroscope showed absorption bands resembling those of carbon monoxide, and reduction upon the addition of ammonium sulphide was delayed and in some cases difficult to obtain, although there was no instance in which reduction was not possible. As to the phenomena occurring in the dendrites of the brain cells, little use can be made of evidence derived from animals which have been killed by the drug, and vivisection laws will not permit the examination of the brain in the various stages of narcosis; but in cats which were killed with ethyl chloride the dendrites were varicose, and had in many instances lost their gemmules.

[I am indebted to Mr. D. Hughes, M.P.S., of Messrs. Buxton & Co., for help in the examination of the blood.]

